# Adaptation of Soil Fungal Community Structure and Assembly to Long- Versus Short-Term Nitrogen Addition in a Tropical Forest

**DOI:** 10.3389/fmicb.2021.689674

**Published:** 2021-08-25

**Authors:** Jinhong He, Shuo Jiao, Xiangping Tan, Hui Wei, Xiaomin Ma, Yanxia Nie, Juxiu Liu, Xiankai Lu, Jiangming Mo, Weijun Shen

**Affiliations:** ^1^Center for Ecological and Environmental Sciences, South China Botanical Garden, Chinese Academy of Sciences, Guangzhou, China; ^2^State Key Laboratory of Crop Stress Biology in Arid Areas, Shanxi Key Laboratory of Agricultural and Environmental Microbiology, College of Life Sciences, Northwest A&F University, Yangling, China; ^3^Department of Ecology, College of Natural Resources and Environment, South China Agricultural University, Guangzhou, China; ^4^College of Forestry, Guangxi University, Nanning, China

**Keywords:** nitrogen addition, fungal community, stochastic processes, deterministic processes, tropical forest

## Abstract

Soil fungi play critical roles in ecosystem processes and are sensitive to global changes. Elevated atmospheric nitrogen (N) deposition has been well documented to impact on fungal diversity and community composition, but how the fungal community assembly responds to the duration effects of experimental N addition remains poorly understood. Here, we aimed to investigate the soil fungal community variations and assembly processes under short- (2 years) versus long-term (13 years) exogenous N addition (∼100 kg N ha^–1^ yr^–1^) in a N-rich tropical forest of China. We observed that short-term N addition significantly increased fungal taxonomic and phylogenetic α-diversity and shifted fungal community composition with significant increases in the relative abundance of *Ascomycota* and decreases in that of *Basidiomycota*. Short-term N addition also significantly increased the relative abundance of saprotrophic fungi and decreased that of ectomycorrhizal fungi. However, unremarkable effects on these indices were found under long-term N addition. The variations of fungal α-diversity, community composition, and the relative abundance of major phyla, genera, and functional guilds were mainly correlated with soil pH and NO_3_^–^–N concentration, and these correlations were much stronger under short-term than long-term N addition. The results of null, neutral community models and the normalized stochasticity ratio (*NST*) index consistently revealed that stochastic processes played predominant roles in the assembly of soil fungal community in the tropical forest, and the relative contribution of stochastic processes was significantly increased by short-term N addition. These findings highlighted that the responses of fungal community to N addition were duration-dependent, i.e., fungal community structure and assembly would be sensitive to short-term N addition but become adaptive to long-term N enrichment.

## Introduction

As one of the major global change drivers, reactive nitrogen (N) from fertilizer application and fossil fuel combustion has increased by 120% over the past few decades (Galloway et al., 2004) and is expected to increase or at least not decrease rapidly in the near future (Yu et al., 2019; Wen et al., 2020). The excessive loading of reactive N has been found to have profound influences on terrestrial ecosystems, such as the reduction of forest soil respiration (Janssens et al., 2010), the attenuation of plant–microbe interactions (Wei et al., 2013), and the alteration of soil biogeochemical cycles (Lu et al., 2011). Fungi play vital roles in the structural and functional dynamics of terrestrial ecosystems. For instance, saprotrophic fungi are the primary decomposers of plant litter and soil organic matter due to their relatively higher capability to degrade cellulose and lignin (Baldrian and Valášková, 2008; Schneider et al., 2012), and mycorrhizal fungi can shape plant community dynamics by affecting seed dispersal, seedling establishment, and intra-/inter-specific interactions (Tedersoo et al., 2020). Previous studies suggested that experimental N addition or fertilization generally shifts the fungal community composition directly by affecting their nutrient supply and indirectly by altering edaphic conditions (Yang Y. et al., 2020; Cui et al., 2021). Different fungal guilds may exhibit different responses to N enrichment. For instance, the relative abundance and diversity of mycorrhizal fungi decreased, whereas that of saprotrophic fungi increased with experimental N addition (Weese et al., 2015; Morrison et al., 2016), thus resulting in the shifts of community composition. Therefore, knowledge of how soil fungal communities respond to N addition is critical for predicting ecosystem responses and for managing plant–fungi interactions under the context of global environmental changes and maintaining sustainable agriculture and forestry (Bissett et al., 2013; Dessaux et al., 2016).

More often, the effects of N enrichment on fungal biomass and diversity are related to the N rates and duration of application (Yang et al., 2019). Previous researches showed that short-term exogenous N enrichment could reduce or increase soil fungal community diversity (Zhang H. et al., 2018; Chen et al., 2020; Zhao et al., 2020), but other studies indicated that fungal communities had the potential to adapt to the long-term N addition (Entwistle et al., 2013; Freedman et al., 2015; Yan et al., 2021). Microorganisms are known for their capabilities of resistance and resilience to perturbations, but it should be noted that microbial communities are generally sensitive to environmental changes and can recover from disturbance across the time scales of days to years (Allison and Martiny, 2008; Li et al., 2014). However, we have limited knowledge on the duration effects of N addition, mainly because molecular approaches characterizing microbial diversity were not available for early long-term N addition experiments. Understanding the duration effects of exogenous N enrichment is therefore helpful for predicting the dynamic responses of fungal communities to future environmental changes.

It is now widely recognized that microbial community assembly is often determined by both deterministic and stochastic processes (Caruso et al., 2011; Nemergut et al., 2013; Zhao Z. et al., 2019). The deterministic processes mainly include selection via environmental filtering and biotic interactions (He C. et al., 2021; He Q. et al., 2021). The stochastic processes consider that microbial species dynamics are controlled by stochastic dispersal, ecological drift, and diversification (Vellend, 2010; Nemergut et al., 2013). Nitrogen enrichment can affect the processes underpinning fungal community assembly, but inconsistent results have been reported. For example, stochastic processes dominate under the fertilization treatment in cropland ecosystems (Shi et al., 2019; Li et al., 2021), whereas deterministic processes were found to have a larger relative importance with fertilization in plantation and grassland ecosystems (Chen et al., 2017; Guo et al., 2020). Moreover, N fertilization resulted in tightened network associations among fungal, bacterial, and protist communities in diverse agricultural soils (Zhao Z. et al., 2019), indicating an increased importance of deterministic biotic interactions in shaping microbiome. Contrastingly, N fertilization did not change the fungal community networks in a temperate grassland soil (Chen et al., 2020). The discrepancies of fungal assembly processes response to N enrichment may be ascribed to the N application rate, duration, ecosystem type, and fungal categories of analysis; however, existing studies were mainly carried out in cropland and grassland ecosystems. It remains of continued relevance to understand how the relative contributions of stochastic versus deterministic processes influence fungal community assembly under N enrichment in forest ecosystems.

In this study, we took advantage of an existing long-term (∼13 years) N addition experiment in a N-rich but phosphorus (P)-deficient tropical forest (Mo et al., 2006), and initiated a new N addition experiment [hereafter the short-term (∼2 years) experiment] beside the long-term N addition experiment (∼2 km away) with similar plant community composition and edaphic conditions (Han et al., 2018). A previous study based on the two experiments revealed that associated functional microbes of N transformation could adapt to long-term N addition to prevent N losses (Han et al., 2018). For this study, we took soil samples from the two experimental sites, and assessed the fungal diversity and community composition through Illumina Hiseq sequencing of internal transcribed spacer (ITS) region. Additionally, we used the null-based statistical framework, neutral community model, and the normalized stochasticity ratio (*NST*) index to quantify the relative contributions of deterministic and stochastic processes in shaping the fungal community. Accordingly, we hypothesized that (i) the short-term N addition would impose significant impacts on soil fungal diversity and composition, and the effects of long-term N addition would be slight; (ii) stochastic processes would dominate at the short-term site since it improves soil N availability for fungi, whereas deterministic processes would be prevalent at the long-term site since long-term excessive N inputs would create a stressful environment for fungal community (Wang et al., 2018); (iii) both the short- and long-term N additions would change the network structures of the soil fungal community, and a more clustered network structure would be observed at the long-term site since it creates a more stressful environment that selects more adaptive fungal guilds.

## Materials and Methods

### Site Description

The long- and short-term N addition experiments were established in Dinghushan Biosphere Reserve (DHSBR) in the city of Zhaoqing, Guangdong province of south China (112°10′ E, 23°10′ N). The reserve lies in a tropical moist forest region and covers about 1,155 ha with a monsoon climate. The mean annual precipitation is 1,748 mm, mainly concentrated from April to September, with only a small part falling from October to March. The mean annual temperature is 21.0°C, with a monthly average temperature ranging from 12.6°C in January to 28.0°C in July. The atmospheric N deposition in the reserve had reached 49.78 kg N ha^–1^ yr^–1^ in 2015–2016 (Zhou et al., 2018). The forest type is evergreen broadleaf forest and it has been characterized as N-rich but P-limited in previous studies (Mo et al., 2006; Lu et al., 2018). The dominant tree species included *Castanopsis chinensis* Hance, *Machilus chinensis* (Champ. Ex Benth.) Hemsl., *Schima superba* Chardn. et Champ., *Cryptocarya chinensis* (Hance) Hemsl., *Cryptocarya concinna* Hance, and *Randia canthioides* Champion ex Bentham. The forest ages are approximately 400 and 100 years for the long- and short-term sites, respectively.

### Experimental Design and Soil Sampling

The long-term experiment was established in 2002, and N addition started in July 2003 with a monthly spraying ammonium nitrate (NH_4_NO_3_) solution (Mo et al., 2006). Three 10 × 20 m^2^ plots were assigned for N addition (N; ambient + 100 kg N ha^–1^ yr^–1^), and three paired plots were designed as control (C; ambient N deposition). For each N addition plot, the required NH_4_NO_3_ was weighed, dissolved in 20 L of water, and sprayed evenly below the canopy using a backpack sprayer. Control plots received 20 L of water, which is equivalent to an increase of 1.2 mm yr^–1^ precipitation. The short-term experiment was established in October 2013; N addition began in September 2014, with 0 and 105 kg N ha^–1^ yr^–1^ NH_4_NO_3_ for control (C) and N addition (N) treatments, respectively. A total of six (three replicates per treatment × two treatments) randomly scattered plots (15 m × 15 m in area) were set up. The required NH_4_NO_3_ was dissolved in 30 L water and sprayed below the canopy monthly in each N addition plot. The control plots received an equivalent volume of water only, which corresponded to an extra 1.6 mm of precipitation that occurred each year.

Surface soil samples (0–20 cm) were collected four times from each plot in the wet (July 2015 and 2016) and dry (January 2016 and 2017) seasons. Within each plot, samples were randomly taken using a soil core (Φ 5 cm) at six spots to form a composite sample for analysis, which resulted in 24 soil samples for each site in the 2 years. Visible roots and plant residues were removed from all samples, which were then sieved through a 2-mm mesh. The sieved samples were divided into two groups. One group was used to determine soil properties, and the other was kept at −80°C for subsequent DNA extraction and microbial community analyses.

### Measurement of Soil Properties

Soil water content (SWC) was measured gravimetrically using 10 g of fresh soil by oven-drying at 105°C for 24 h. Soil pH was determined in a 1:2.5 air-dried soil:water suspension (w:v) using a pH meter (F-71G, LAQUA, HORIBA, Japan). Soil ammonium N (NH_4_^+^-N) and nitrate N (NO_3_^–^-N) were extracted by 1 M KCl from 20 g fresh soil samples and detected using the indophenol blue and dual wavelength (220 nm and 275 nm) colorimetric methods (UV-6000, China), respectively. Soil total organic carbon (TOC) was analyzed using an external heating method with concentrated sulfuric acid and potassium dichromate (H_2_SO_4_-K_2_Cr_2_O_7_). To examine the contents of soil total N (TN) and total phosphorus (TP), semi-micro Kjeldahl digestion was carried out followed by indophenol blue and molybdenum antimony blue colorimetric methods, respectively. Soil dissolved organic carbon (DOC) and N (DON) were extracted with 0.5 M K_2_SO_4_ solution and measured using a TOC analyzer (Shimadzu TOC-VCSH Analyzer, Kyoto, Japan).

### Soil DNA Extraction and Sequencing

Total DNA from soil was extracted using the PowerSoil^®^ DNA Isolation Kit (MoBio Laboratories Inc., Carlsbad, CA, United States) according to the manufacturer’s instructions. The extracted DNA was examined for quantity and quality using a Nanodrop 2.0 spectrophotometer (Thermo Fisher Scientific, Carlsbad, CA, United States). The paired primers ITS3-F (5′-GCATCGATGAAGAACGCAGC-3′) and ITS4-R (5′-TCCTCCGCTTATTGATATGC-3′) were used to amplify the ITS2 region in the fungal rRNA operon (White et al., 1990). The PCR and tag-encoded high-throughput sequencing of the ITS2 were performed using the Illumina HiSeq platform (PE 250) (Guangdong Magigene Biotechnology Co., Ltd., Guangzhou, China).

### Sequence Processing and Bioinformatics

The raw sequences were cleaned to ensure high-quality data; the reads with short sequences (<200 bp), barcodes, and poly bases etc. were removed using the Fastp^1^ (V0.14.1) and cutadapt^2^ (Martin, 2011; Chen et al., 2018). The high-quality paired-end reads were then merged by USEARCH^3^ (V10.0.240) with a minimal overlap of 16 bp. Then the sequences were split into operational taxonomic units (OTUs) through the UPARSE pipeline, which performed chimera filtering and OTU clustering simultaneously, based on a 3% dissimilarity level. OTUs with fewer than two sequences were removed, and the representative sequence of each OTU was assigned to fungal taxonomic lineages by comparison with the UNITE database^4^ (V7.1). All the non-fungal OTUs were removed before downstream analyses. Eventually, to compensate for the uneven sequencing efforts of different samples, the OTU table was randomly subsampled to obtain the same sequence number (61,292) for all the fungal data samples using the *vegan* package in R^5^ (V3.6.1).

We classified the fungal OTUs into functional groups according to the FUNGuild database (Nguyen et al., 2016). The taxa with confidence levels “highly probable” or “probable” and unique trophic guilds which belong to saprotrophic fungi, pathogenic fungi, or ectomycorrhizal (EcM) fungi were selected for further analyses.

### Phylogenetic Community Assembly

The phylogenetic signals were assessed for the control and N addition treatments at each site to test whether fungal community responses to N addition was phylogenetically conserved. Phylogenetic correlogram was applied to test the phylogenetic signals and measured by the “mantel.correlog” function in the *vegan* package. The assembly processes of fungal community were evaluated using a null model analysis (Stegen et al., 2012, 2015; Dini-Andreote et al., 2015). Accordingly, ecological processes, which were classified into deterministic processes (e.g., variable and homogeneous selections) or stochastic processes (dispersal limitation, homogenizing dispersal, and undominated), were evaluated by β-nearest taxon index (βNTI) in combination with Raup-Crick metric (Bray–Curtis-based Raup-Crick, RC_bray_) (Stegen et al., 2012, 2013, 2015; Zhou and Ning, 2017). Based on Stegen et al. (2012), βNTI > 2 (or βNTI < −2) between a pair of samples means the significantly higher (lower) phylogenetic turnover than expected (null distribution), indicating the predominance of variable selection (or homogeneous selection). Subsequently, the RC_bray_ was calculated to estimate the pairwise comparisons with | βNTI| < 2. The percentage of homogeneous dispersal was quantified as the fraction of pairwise comparisons with | βNTI| < 2 and RC_Bray_ < −0.95, while the dispersal limitation was quantified as the fraction of the pairwise comparisons with | βNTI| < 2 and RC_bray_ > 0.95. Finally, the remaining fractions (| βNTI| < 2 and | RC_bray_| < 0.95) were treated as undominated processes (Stegen et al., 2015; Zhou and Ning, 2017).

To further quantify the contributions of stochastic (neutral) processes to fungal community structure under different N treatments, a normalized stochasticity ratio (*NST*) and a Sloan neutral community model were applied (Sloan et al., 2006; Ning et al., 2019). The *NST*, an index developed with 50% as the boundary point between more deterministic (<50%) and more stochastic (>50%) assembly, was quantified using a pipeline based on the phylogenetic distance^6^. The Sloan neutral community model was fitted using the R script reported by Barnes et al. (2016). *R*^2^ indicates the fit of the parameter based on nonlinear least-squares fitting.

### Co-association Networks of Fungal Community

Microbial linkage is one of the main drivers that contribute to the deterministic process of community assembly. To estimate the interspecies linkage within the fungal community across the control and N treatments at each site, co-association networks were constructed based on the Random Matrix Theory (RMT) using the Molecular Ecological Network Analysis Pipeline^7^ (Deng et al., 2012). Briefly, fungal OTU table was square root-transformed and then split into four sub-datasets according to duration and rate of N addition. For each sub-dataset, only those nodes detected in more than half of the total samples (majority rule) were kept for subsequent network construction. More information on theories, algorithms, pipeline structures, and procedures can be found in references (Zhou et al., 2011; Deng et al., 2012). Network visualization was conducted using the interactive platform Gephi (WebAtlas, Paris, France). The “randomize the network structure and then calculate network” function was run to compare the properties of the empirical and randomly generated networks. The topological roles of each node in the network were determined by the threshold values of within-module connectivity (*Zi*) and among-module connectivity (*Pi*) as proposed by Guimerà and Amaral (2005) in each network. Network hubs (*Zi* > 2.5 and *Pi* > 0.62), module hubs (*Zi* > 2.5 and *Pi* < 0.62), and connectors (*Zi* < 2.5 and *Pi* > 0.62) were termed keystone network topological features, and thus the OTUs associated with these nodes were defined as keystone species (Zhou et al., 2011; Deng et al., 2012).

### Statistical Analyses

The phylogenetic α-diversity was calculated with the *picante* package in R. The taxonomic α-diversity (Richness and Shannon diversity index) was calculated by USEARCH. Three-way analysis of variance (ANOVA) was used to evaluate the effects of the duration, rate, and season of N addition and their interactions on fungal taxonomic and phylogenetic α-diversity. As the results showed that season had no significant effect on fungal α-diversity, we considered the seasons as the replicates for downstairs analysis (Supplementary Table 1). Therefore, two-way ANOVA was performed to analyze the effects of duration, rate of N addition and their interactions on soil properties, fungal α-diversity, and the relative abundance of major phyla, genera, and functional guilds. Student’s *t*-test was used to assess the differences of aforementioned parameters between the control and N addition treatments in the short- and long-term sites, respectively. Before two-way ANOVA and Student’s *t*-test were performed, the data were checked for normality (Shapiro test) and homoscedasticity (Bartlett test). If the datasets were not normal or homoscedastic, they were subjected to the Box-Cox transformation, ensuring that they met the assumptions of these tests. Linear regression models were used to express the correlations between soil properties and community variables, such as α-diversity and relative abundance of major phyla, genera, and functional guilds.

The multivariate permutational analysis of variance (PERMANOVA) was conducted to analyze the variances in taxonomic (based on Bray–Curtis distance) and phylogenetic (based on beta mean nearest taxon distance; βMNTD) community composition and visualized by non-metric multidimensional scaling (NMDS). Analysis of similarities (ANOSIM) with 999 permutations based on Bray–Curtis dissimilarity and βMNTD was conducted to identify differences in OTU composition within and between treatments. The potential relationships between environmental variables and fungal taxonomic and phylogenetic community composition were assessed using constrained analysis of principal coordinates (CAP) analysis based on the “capscale” function of the *vegan* package in R. The significantly environmental factors were selected using “envfit” function of the *vegan* package with 999 random permutations. The variables that displayed significant effects (*p* < 0.05) were included in the ordination plot. To help reveal the variation in species composition of the fungal community, the Levins’ niche breadth (*Bcom*) index (Levins, 1968) was determined using the “niche.width” function in the *spaa* package in R. All the statistical analyses were performed in R.

## Results

### Responses of Soil Physicochemical Properties to N Addition

Of all the soil physicochemical parameters measured, only the soil pH and NO_3_^–^–N concentration were significantly affected by N addition (Student’s *t*-test, *p* < 0.05; Supplementary Table 2). Specifically, soils from both sites were strongly acidic with an average pH of 3.93, and N addition significantly decreased the soil pH by 0.1 unit at both sites (*p* < 0.05). Nitrogen addition also significantly increased the soil NO_3_^–^–N concentration by 56.11% at the short-term site, while only slightly (by 33.14%, *p* > 0.05) increased at the long-term site.

### Responses of Fungal Diversity and Community Composition to N Addition

**FIGURE 1:**
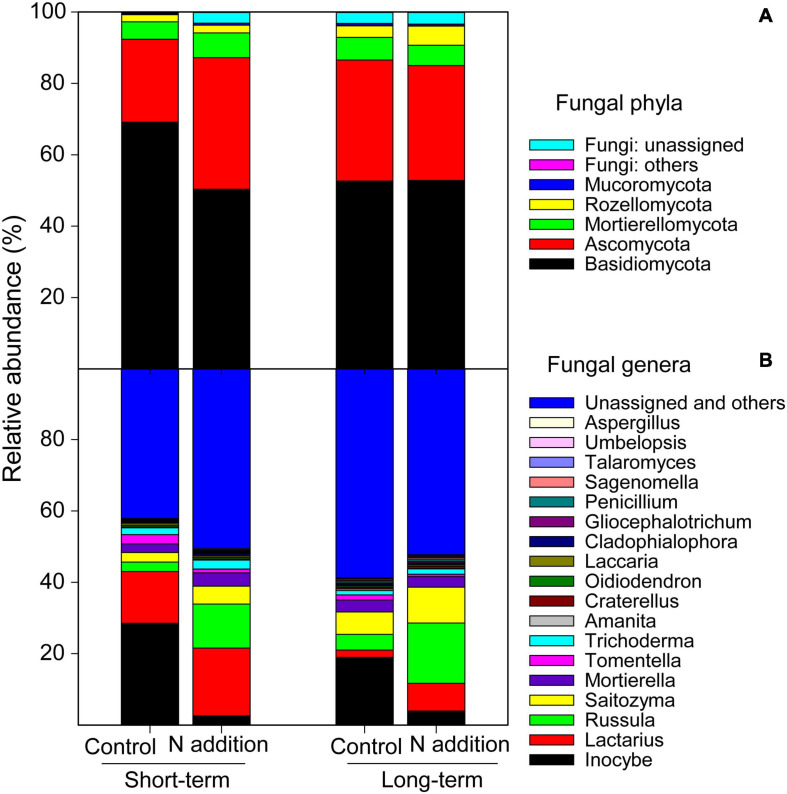
Relative abundance of major soil fungal phyla **(A)** and genera **(B)** at the short- and long-term N addition sites. Phyla and genera with relative abundance >0.2% are included.

After a series of qualified and filtered steps, 2,942,016 sequences were retained and clustered into 4,672 fungal OTUs. Of all the samples, a total of 96.8% of the sequences could be assigned into 11 phyla and 261 genera (Figure 1). At the phylum level, *Basidiomycota* was predominant, followed by *Ascomycota*. The relative abundance of both phyla was significantly impacted by N addition and differed significantly between the long- and short-term sites (*p* < 0.05; the insert text in Supplementary Figures 1A,B). At the short-term site, N addition significantly decreased the relative abundance of *Basidiomycota* by 27.19%, but significantly increased that of *Ascomycota* by 58.82% (Supplementary Figures 1A,B). However, the relative abundances of these phyla were not significantly affected by the long-term N addition (*p* > 0.05). At the genera level, the four dominant taxa were *Inocybe*, *Lactarius*, *Russula*, and *Saitozyma* representing 13.5%, 10.8%, 9.1%, and 6.0% of all sequences, respectively (Figure 1B and Supplementary Figures 1C–F). At both sites, the N addition decreased the relative abundance of *Inocybe* (by 90.93% and 79.32% at short- and long-term sites, respectively) but increased that of *Russula* (by 368.04% and 283.98% at short- and long-term sites, respectively), *Lactarius* (by 30.47% and 282.31% at short- and long-term sites, respectively), and *Saitozyma* (by 87.13% and 60.44% at short- and long-term sites, respectively). At the functional guild level, approximately 43.4% sequences were categorized into EcM fungi, and the short-term N addition significantly decreased the relative abundance of this group by 26.13% (Supplementary Figure 2A). Nevertheless, only 3.5% of sequences were classified as saprotrophic fungi, and the short-term N addition significantly increased the relative abundance of this group (Supplementary Figure 2B).

**FIGURE 2:**
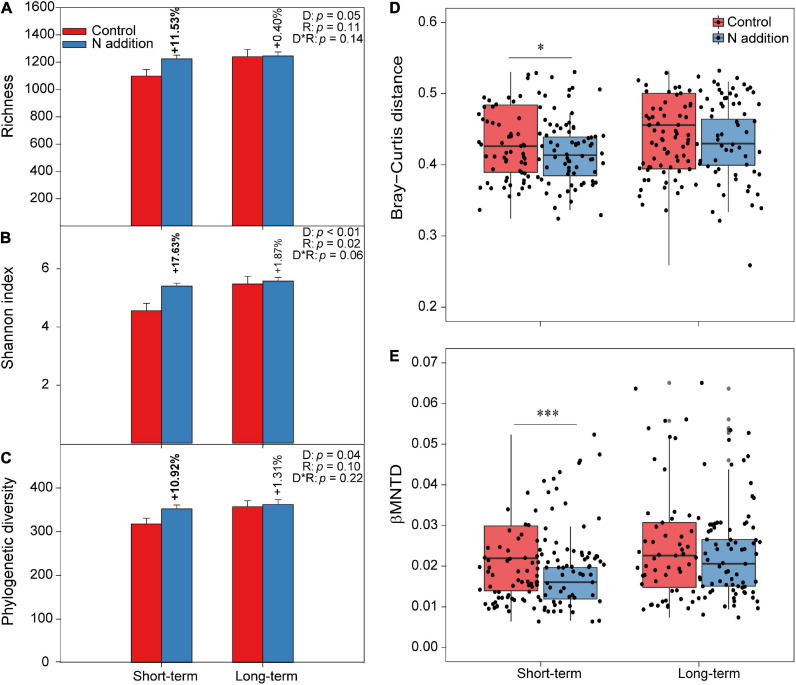
The varations of fungal α-diversity and community composition under short- and long-term N additions. **(A–C)** The varaitions of fungal α-diversity under N addition are indicated by Student’s *t*-test at the short- and long-term sites, respectively. The bold numbers denote the significantly differences (*p* < 0.05) of fungal α-diversity between control and N addition at each site. The insert texts indicate the effects of duration (D), rate (R) of N addition, and their interactions on fungal α-diversity detected by two-way ANOVA. **(D,E)** The boxplots show community dissimilarities between control and N addition at the short- and long-term sites based on Bray–Curtis distance and βMNTD, respectively (^∗^*p* < 0.05; ^∗∗∗^*p* < 0.001; Wilcoxon rank-sum test).

The two-way ANOVA indicated that the fungal taxonomical and phylogenetic α-diversity was significantly influenced by the duration of N addition (*p* < 0.05; the insert text in Figures 2A–C). Specifically, the three fungal α-diversity indices (Richness, Shannon, and phylogenetic diversity) increased significantly (by more than 10%) at the short-term N addition site (*p* < 0.05), while they were not significantly altered by the N addition at the long-term site. Moreover, the PERMANOVA indicated that the duration, rate of N addition, and their interactions significantly shaped the fungal community composition (*p* < 0.05; the insert text in the top right corner of Supplementary Figure 3A). Specifically, the soil fungal community could be distinctly separated by short- and long-term N additions based on the Bray–Curtis distance metric by NMDS plots. In addition, the dissimilarity of the soil fungal community was significantly decreased by short-term N addition (Figure 2D). When considering the variation in phylogenetic community composition (based on βMNTD), the duration of N addition significantly changed the fungal phylogenetic community composition; N addition significantly decreased βMNTD at the short-term site, while significant changes were not found at the long-term site (Figure 2E and Supplementary Figure 3B). Moreover, fungal community in N addition soils had a higher habitat niche breadth than that in control soils at both sites (Supplementary Figure 4).

### Factors Affecting Fungal Diversity and Community Composition Under N Addition

**FIGURE 3:**
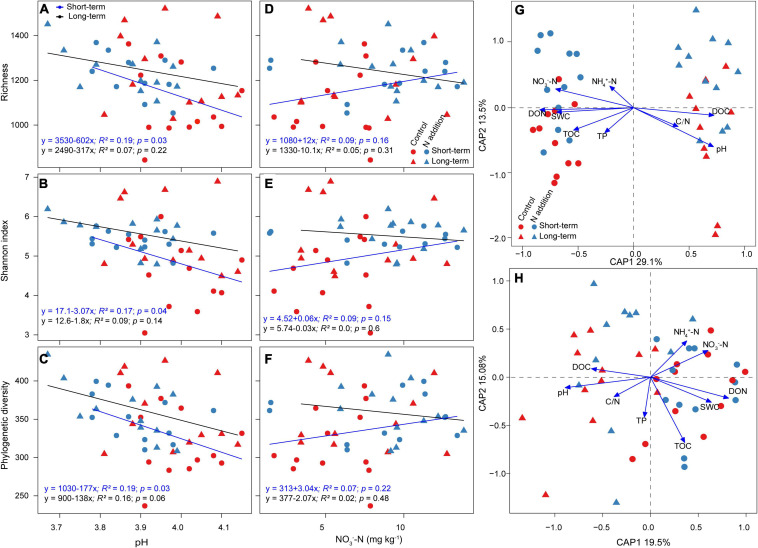
Factors affecting fungal α-diversity and community composition. **(A–F)** Relationships between fungal α-diversity and soil pH and NO_3_^–^ –N concentration are revealed by linear regression model. **(G,H)** CAP analysis shows the community dissimilarities based on Bray–Curtis distance and βMNTD against environmental variables among samples. SWC, soil water content (%); NH_4_^+^–N, ammonium N (mg kg^–1^); NO_3_^–^–N, nitrate N (mg kg^–1^); TOC, total organic carbon (%); TN, total N (%); C/N, total organic carbon/total N; TP, total phosphorus (%); DOC, dissolved organic carbon (mg kg^–1^); DON, dissolved organic N (mg kg^–1^).

The linear regression indicated that the relative abundance of major functional guilds, phyla, and genera was mainly correlated with soil pH and NO_3_^–^–N concentration, and the correlations were much stronger at the short-term than long-term site (Supplementary Figures 5, 6). The relative abundance of EcM fungi and *Basidiomycota* was significantly positively correlated with soil pH, and that of saprotrophic fungi and *Ascomycota* was significantly negatively correlated with pH at the short-term site, while these correlations were not significant at the long-term site. The soil pH, not NO_3_^–^–N concentration, was the dominant driver to predict the variations of fungal richness, Shannon index, and phylogenetic diversity (Figures 3A–F). The aforementioned α-diversity indices were negatively correlated with soil pH, and the correlations were more significant at the short-term than the long-term site. The CAP analysis also revealed that soil pH was the most important variable for the shifts of fungal taxonomic and phylogenetic community composition, and soil nutrients (such as DON, DOC, and NO_3_^–^–N) were also important for fungal community composition (Figures 3G,H and Supplementary Table 3).

### Responses of Fungal Assembly Processes to N Addition

**FIGURE 4:**
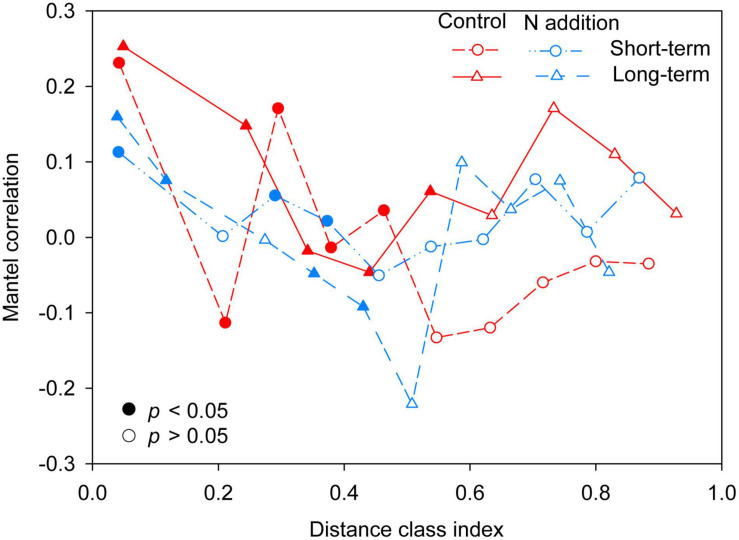
Phylogenetic mantel correlogram between the Euclidean distance matrix of OTU niche values and phylogenetic distance matrix. Solid and open symbols denote significant (*p* < 0.05) and non-significant (*p* > 0.05) correlations (phylogenetic signal), respectively.

**FIGURE 5:**
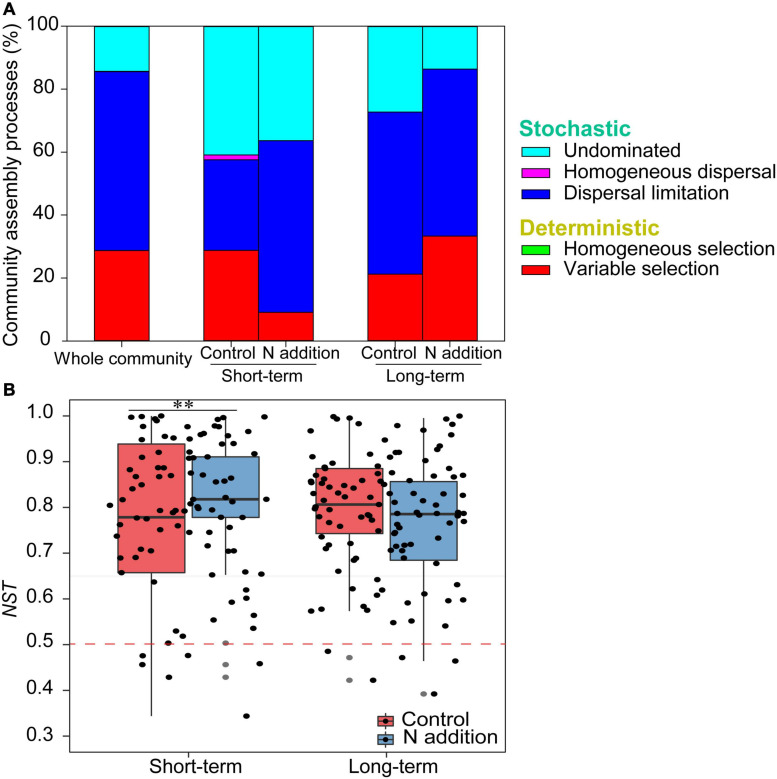
The assembly processes in shaping fungal community. **(A)** The relative contributions (%) of the five community assembly processes. **(B)** Boxplot shows the variation in *NST* under control and N addition at the short- and long-term sites (^∗∗^*p* < 0.01; Wilcoxon rank-sum test).

The mantel correlogram showed significant phylogenetic signals across relatively short phylogenetic distances for the four sub-communities (*p* < 0.05, Figure 4), indicating that the fungal community in response to N enrichment are phylogenetically conserved and that the closely related species in the fungal community exhibited more similar ecological preferences to the environmental variables. Null model analysis revealed that stochastic processes mainly governed fungal community dynamics in the whole community and the four sub-communities (Figure 5A). In the sub-communities, the relative contribution of stochasticity was increased by short-term N addition from 71.2% (control) to 90.9% (N addition), while it was slightly decreased by long-term N addition from 78.8% (control) to 66.7% (N addition). The *NST* results also showed the fungal community was predominately governed by stochastic processes (*NST* = 81.8% and 82.7% for the control treatment at the short- and long-term sites and 89.0% and 79.2% for the N addition treatment at the short- and long-term sites, respectively). Specifically, the *NST* increased significantly by short-term N addition (*p* < 0.01) but decreased slightly by long-term N addition (Figure 5B). The neutral model explained a large fraction (*R*^2^ > 0.5) of the variability in the occurrence frequency of the fungal community, with more than 80% of species having frequencies within predicted ranges (Supplementary Figure 7).

### Responses of Fungal Co-association Patterns to N Addition

**FIGURE 6:**
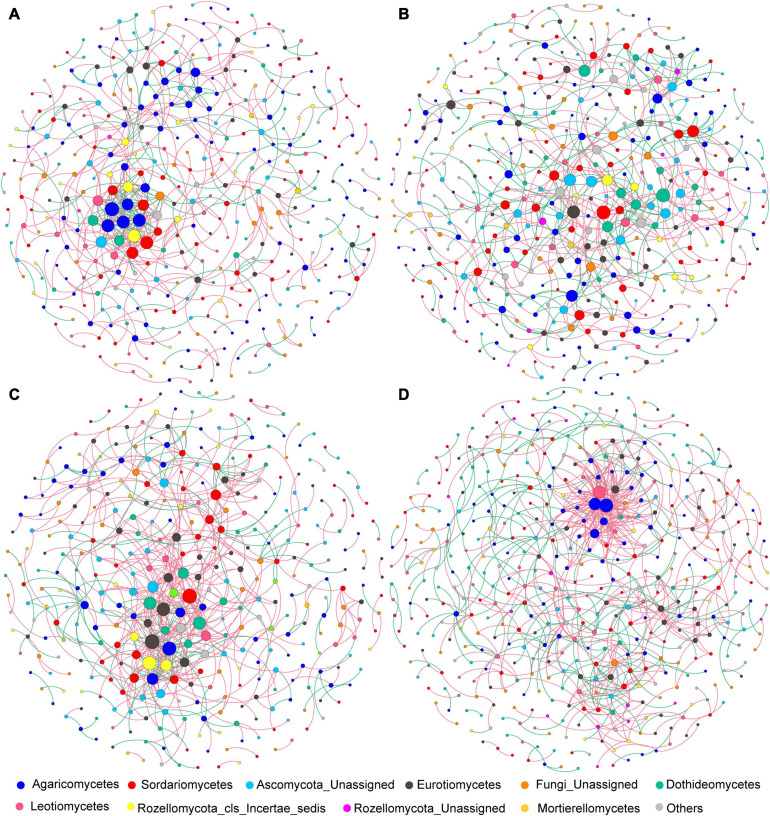
**(A–D)** Co-association patterns of OTUs of fungal community in control and N addition treatments at the short- and long-term sites, respectively. The size of each node is proportional to the number of connections (i.e., degree). The red and green edges indicate positive and negative associations between two individual nodes, respectively.

After data filtration, the remaining 405, 451, 369, and 461 OTUs were used to construct networks for the four sub-communities (Table 1). The networks showed topological properties of small-world, scale-free and modularity, and were significantly different from randomly generated networks. The N addition slightly weakened the inter-connections among fungal species at both sites (Figure 6 and Table 1), with a lower average clustering coefficient (avgCC) and average connectivity degree (avgK), and a higher average path length (APL) and centralization of betweenness (CB). These results indicated that the networks were less clustered in the N addition treatments. Additionally, positive correlations were predominant in all networks and the number of positive correlations was decreased by N addition at both sites.

**TABLE 1:** Detailed topological properties of the empirical and random networks of fungal community in soils under control and N addition at the short- and long-term sites, respectively.

**Network index**	**Short-term site**		**Long-term site**	
	**Control**	**N addition**	**Control**	**N addition**
*Empirical networks*				
Total nodes	405	451	369	461
Total links	596	638	630	770
Positive links (%)	65.94	55.96	72.06	65.58
Negative links (%)	34.06	44.04	27.94	34.42
Similarity threshold (St)	0.81	0.81	0.85	0.82
*R*^2^ of power-law	0.848	0.894	0.91	0.805
Average connectivity (*avgK*)	2.943	2.829	3.415	3.341
Average clustering coefficient (*avgCC*)	0.159	0.149	0.152	0.122
Average path distance (GD)	7.55	8.589	5.803	7.662
Modularity (module no.)	0.792 (50)	0.825 (48)	0.678 (49)	0.781 (40)
Diameter	21	22	14	24
Density (D)	0.007	0.006	0.009	0.007
Geodesic efficiency (E)	0.171	0.15	0.214	0.171
Harmonic geodesic distance (HD)	5.861	6.687	4.679	5.85
Maximal degree	20	13	21	43
Centralization of degree (CD)	0.042	0.023	0.048	0.087
Centralization of betweenness (CB)	0.081	0.159	0.083	0.169
Centralization of stress centrality (CS)	0.357	2.258	1.133	3.413
*Random network*				
Avg. clustering coefficient	0.015 (0.005)	0.007 (0.003)	0.020 (0.005)	0.021 (0.005)
Average path distance	4.619 (0.081)	5.309 (0.077)	4.202 (0.056)	4.332 (0.053)
Avg. modularity	0.625 (0.007)	0.658 (0.006)	0.555 (0.007)	0.573 (0.006)

**FIGURE 7:**
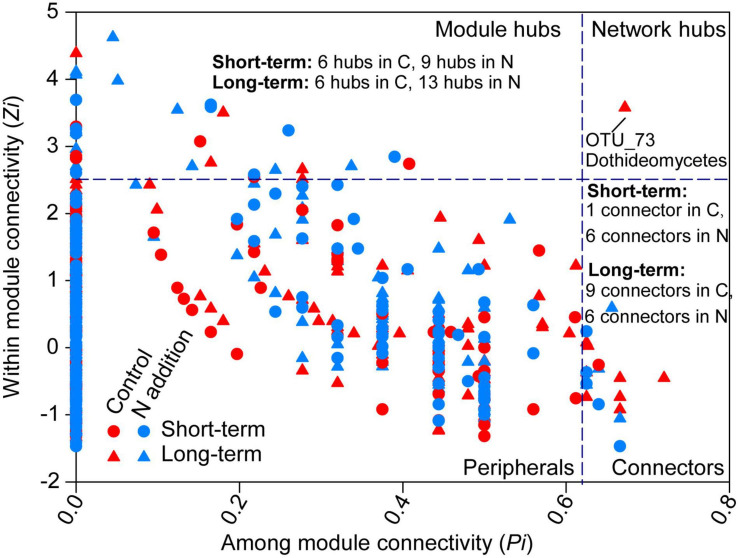
Classification of nodes to identify putative keystone species within the networks. The topological role of each OTU is determined according to the scatter plot of within-module connectivity (*Zi*) and among-module connectivity (*Pi*). Module hubs have *Zi* > 2.5, whereas connectors have *Pi* > 0.62. The number of module hubs and connectors is presented on the plot and the network hub is labeled with the OTU number. The abbreviations of C and N are defined as control and N addition, respectively. Detailed taxonomic information for module hubs and connectors is shown in Supplementary Tables 4–7.

The topological roles of the OTUs identified in the networks were shown as a *Zi-Pi* plot in Figure 7. The majority of the OTUs were peripherals (also regarded as specialist) with most of their links being inside their modules. A total of 8, 15, 16, and 19 OTUs were classified as module hubs, collectors, and network hubs (putative keystone species) for the four sub-networks (Figure 7 and Supplementary Tables 4–7). The networks in the control and N addition treatments at the short-term site shared one putative keystone species (OTU140; *Eurotiomycetes*, *Ascomycota*), while the networks at the long-term site did not share any keystone species. Most putative keystone species belonged to the classes *Eurotiomycetes*, *Agaricomycetes*, *Sordariomycetes*, *Dothideomycetes*, *Leotiomycetes*, *Mortierellomycetes*, and *Tremellomycetes*.

## Discussion

### Soil Fungal Diversity and Community Composition Were More Sensitive to Short-Term N Addition

In this study, we used the method of substitution of space for time to assess the duration effects of N addition on the soil fungal community. We found that the soil fungal α-diversity was enhanced by N addition (∼100 kg N ha^–1^ yr^–1^) at both the short- and long-term sites (Figures 2A–C), indicating that soil fungi would tend to respond to environmental disturbance by enrichment and domestication (Shi C. et al., 2020; Shi J. et al., 2020). The increased fungal α-diversity at both sites may be explained by the following reasons: (i) N addition enhanced the plant net primary productivity of the tropical forest (Du and de Vries, 2018; Liang et al., 2020), leading to more plant-derived resources for soil fungal growth; (ii) the rate of N addition (∼100 kg N ha^–1^ yr^–1^) likely caused an intermediate disturbance and led to many vacant ecological niches that facilitate fungal colonization, according to the intermediate disturbance hypothesis (Zhang X. et al., 2018); (iii) the internal pH regulatory mechanism of soil fungi can make their cells maintain a relatively neutral pH (Gross and Robbins, 2000), and thus, the acidic soil induced by N addition could result in more abundant acidophilic or acid-tolerant fungi in the studied forest. However, different response patterns were found in the N-limited temperate and boreal forests, which indicated that soil fungal α-diversity or abundance was decreased or not significantly affected by N addition (Xia et al., 2020; Zhou et al., 2020; He W. et al., 2021). We presume that the discrepancies might originate from the differences in the initial N status of the forest ecosystems.

Additionally, consistent with our first hypothesis, we observed that N addition significantly increased the fungal α-diversity at the short-term site (>10%), but little enhanced at the long-term site (<2%; Figures 2A–C), suggesting that soil fungal species could be sensitive to short-term N addition, and may adapt to long-term N enrichment in the tropical forest. Our results are in line with some of the observations from other forest ecosystems (Entwistle et al., 2013; Zhao et al., 2020). For instance, Zhao et al. (2020) revealed that 5 years of N addition increased the fungal α-diversity in a mixed deciduous forest; while Entwistle et al. (2013) showed there were no significant effects on fungal α-diversity under more than 15 years of N addition. Different response patterns between short- and long-term N additions in the present study could be attributed to the sensitivity of fungal species to N availability. For instance, short-term N addition significantly increased fungal richness (Figure 2A), suggesting that some rare taxa were stimulated and appeared, while the prolonged N addition at the long-term site would form a relatively stable fungal community. Moreover, our results were supported by the resistant and resilient abilities of the soil fungal community to environmental disturbance, which indicated that the fungal community could recover to the original state after decades of exogenous N input (Allison and Martiny, 2008).

Furthermore, N addition also altered the fungal community composition between the short- and long-term sites, and the dissimilarities were much greater under short-term than long-term N addition (Figures 2D,E and Supplementary Figure 3). These dissimilarities of fungal community composition may result from the changes of taxonomic structures. For example, N addition significantly decreased the relative abundance of *Basidiomycota*, and increased that of *Ascomycota* at the short-term site. Additionally, short-term N addition also significantly increased the relative abundance of saprotrophic fungi, and decreased that of EcM fungi. However, the relative abundances of these taxa were not significantly impacted by long-term N addition (Supplementary Figures 1, 2), indicating the duration-dependent effects of N addition on fungal populations. Wang et al. (2019) revealed that N addition (105 kg N ha^–1^ yr^–1^) suppressed the fine root production and turnover at the short-term site, which would reduce the carbon supply to their associated ectomycorrhizas and decrease the EcM colonization (Corrales et al., 2017). The positive effects of N addition on relative abundance of saprotrophic fungi may be supported by the “Gadgil effect,” in which N competition between EcM and saprotrophic fungi could benefit to saprotrophic growth (Gadgil and Gadgil, 1971; Fernandez and Kennedy, 2016). Such shifts in fungal community composition could affect the soil enzymatic activities and litter decomposition and, thus, modulate soil C, N, and P cycling in forests exposed to atmospheric N deposition (Ning et al., 2018). Recent studies demonstrated that soil pH and NO_3_^–^–N concentration were the key drivers influencing fungal community composition (Francioli et al., 2016; Ullah et al., 2019; Wang et al., 2021). The relative abundance of dominant functional guilds, phyla, and genera (Supplementary Figures 5, 6) was significantly positively or negatively correlated with soil pH and NO_3_^–^–N concentration at the short-term site, depending on their preference to N and tolerance to pH, which also proved the soil fungal community to be sensitive to short-term N addition. Additionally, the fungal community in N addition soils had a wider habitat niche breadth than that in control soils at both sites (Supplementary Figure 4), indicating that the fungal community has greater metabolic plasticity and governed less by environmental filtering under N addition (Wu et al., 2018).

### Soil Fungal Community Assembly Was Mainly Shaped by Stochastic Processes

The significant phylogenetic signals were found across relatively short phylogenetic distances (Figure 4), in line with previous studies on fungal community (Isobe et al., 2019; Yang J. et al., 2020). The results suggested that closely related species exhibited more similar ecological preferences across environmental variations, and fungal responses to N addition exhibited phylogenetic conservation.

Revealing the assembly rules of the microbial community is a central issue in microbial ecology (Nemergut et al., 2013). Deterministic and stochastic processes are two dominant themes in the study of microbial community dynamics; therefore, it is critical to quantify the relative contributions of the two processes to community assembly (Stegen et al., 2013; Zhou and Ning, 2017). In the present study, the null model and *NST* analysis consistently showed that stochastic processes were more important than deterministic processes with respect to soil fungal community assembly under control and N treatments at both sites (Figure 5), which were partially consistent with our second hypothesis. We speculated the short-term N addition enriched soil nutrients (e.g., NO_3_^–^–N), widened the habitat niche breadth of the fungal community, and weakened the effects of environmental filtering. However, the long-term N addition did not change the soil properties much (Supplementary Table 2), and led to less habitat heterogeneity. This may make a greater relative importance of stochastic events on community composition. Additionally, the hyphal networks of fungi and the roots of their hosts limit the dispersion of fungal species (i.e., dispersal limitation), since most of the dominant trees in the forest are EcM or arbuscular mycorrhizae (Lu et al., 2018; Gurmesa et al., 2019). Our results support the view that stochastic processes predominate in the fungal community assembly (Shi et al., 2019; Zhao J. et al., 2019; Jiao and Lu, 2020). Furthermore, the neutral community model explained a relatively large fraction (*R*^2^ > 0.5) of fungal community variation, indicating that the fungal community was more influenced by neutral (or stochastic) processes.

### Co-association Patterns of Fungal Community Under Short- and Long-Term N Additions

Microorganisms can form complex linkage networks to coexist by occupying specific ecological niches or responding similarly to environments (Faust and Raes, 2012). The co-association networks can present new insights into the potential linkage and assembly process of microbial communities under environmental disturbance (Barberán et al., 2012). Nitrogen addition generated looser and more random fungal networks at both sites (Figure 6 and Table 1), indicating that the putative linkage between fungal species were weakened by N addition. This result supports our third hypothesis that N addition would alter the fungal network structure. Moreover, network parameters such as *avgK* and *avgCC* were higher in control than N addition, suggesting that higher fungal diversity is not necessarily associated with more complex networks. We speculated that higher fungal diversity in N addition soils could lead to a higher degree of functional redundancy, which provides more opportunities for fungal species to correlate with their neighbors, as microorganisms tend to interact with each other by function or metabolite preference (Levy and Borenstein, 2013; Tu et al., 2020). As a result, N addition would lead to weak interspecies linkage and loose co-association networks of the fungal community. Additionally, positive correlations dominated all the networks (Figure 6), implying that mutual cooperation rather than competitive exclusion played a dominant role in fungal community assembly.

## Conclusion

In this study, we compared the variations and responses of fungal taxonomic and phylogenetic α-diversity and community composition between two nearby field experiments in a tropical forest which had received short- (2 years) and long-term (13 years) simulated N deposition (∼100 kg N ha^–1^ yr^–1^). Our results indicated that fungal α-diversity was significantly increased by the short-term N addition rather than the long-term treatments. Additionally, the variations of fungal α-diversity and community composition were greater in short-term compared to long-term N addition. Soil pH and NO_3_^–^–N concentration were the major factors mediating the variations of fungal α-diversity and community composition. Stochastic processes played predominant roles in the assembly of the soil fungal community under N addition at both sites, and the stochasticity was significantly increased by short-term N addition. Moreover, both short- and long-term N additions slightly loosened the co-association networks of fungal community. Together, our results revealed that the soil fungal community structure would be sensitive to short-term N addition, but become adaptive to long-term N addition. The study will contribute to our understanding of underlying mechanisms that regulate the variations of soil fungal community and facilitate the prediction of fungal responses to the ongoing atmospheric N deposition in tropical forests.

## Data Availability Statement

All raw sequences from this paper have been submitted to the NCBI Sequence Read Archive (SRA) database under the BioProject number PRJNA664903.

## Author Contributions

WS and JH conceived and designed the study, and wrote the manuscript. JH, XT, HW, XM, and YN were responsible for performing the field and laboratory work. JH and SJ analyzed the data. All authors discussed the results, critically reviewed the manuscript, and approved its publication.

## Conflict of Interest

The authors declare that the research was conducted in the absence of any commercial or financial relationships that could be construed as a potential conflict of interest.

## Publisher’s Note

All claims expressed in this article are solely those of the authors and do not necessarily represent those of their affiliated organizations, or those of the publisher, the editors and the reviewers. Any product that may be evaluated in this article, or claim that may be made by its manufacturer, is not guaranteed or endorsed by the publisher.

## Footnotes

1
https://github.com/OpenGene/fastp

2
https://github.com/marcelm/cutadapt/

3
http://www.drive5.com/usearch/

4
http://unite.ut.ee

5
http://www.r-project.org/

6
http://ieg3.rccc.ou.edu:8080/

7
http://ieg4.rccc.ou.edu/mena
