# Supplementary material for: Adaptation of Soil Fungal Community Structure and Assembly to Long- Versus Short-Term Nitrogen Addition in a Tropical Forest
Source: Front Microbiol. 2021 Aug 25;12:689674. doi: 10.3389/fmicb.2021.689674 (PMC8424203; doi:10.3389/fmicb.2021.689674)
Supplement: Supplementary file 1 [file Data_Sheet_1.doc]

**Supplementary materials for**

**Adaptation of soil fungal community structure and assembly to long- versus short-term nitrogen addition in a tropical forest**

Jinhong He ^1^, Shuo Jiao ^2^, Xiangping Tan ^1^, Hui Wei ^3^, Xiaomin Ma ^1^, Yanxia Nie ^1^, Juxiu Liu ^1^, Xiankai Lu ^1^, Jiangming Mo ^1^, Weijun Shen ^4, *^

^1^ Center for Ecological and Environmental Sciences, South China Botanical Garden, Chinese Academy of Sciences, 723 Xingke Rd., Tianhe District, Guangzhou 510650, China

^2^ State Key Laboratory of Crop Stress Biology in Arid Areas, Shanxi Key Laboratory of Agricultural and Environmental Microbiology, College of Life Sciences, Northwest A&F University, Yangling 712100, China

^3^ Department of Ecology, College of Natural Resources and Environment, South China Agricultural University, Guangzhou 510642, China

^4^ College of Forestry, Guangxi University, Nanning 530004, China

* Corresponding author: Dr. Weijun Shen

College of Forestry, Guangxi University

100# Daxue Road, Nanning 530004, Guangxi, China

Tel.: + 86 13977164892

Email: [shenweijun@gxu.edu.cn](mailto:shenweijun@gxu.edu.cn)

**SUPPLEMENTARY TABLES**

**TABLE S1 | The effects of duration (D), rate (R), season (S) of N addition and their interactions on soil fungal taxonomic and phylogenetic α-diversity**

|  | Taxonomic diversity | | | | Phylogenetic diversity | |
| --- | --- | --- | --- | --- | --- | --- |
|  | Richness | | Shannon index | |  | |
|  | *F* | *p* | *F* | *p* | *F* | *p* |
| Duration (D) | 3.83 | 0.06 | **7.90** | **<0.01** | **4.29** | **0.045** |
| Rate (R) | 2.46 | 0.12 | **4.60** | **0.04** | 2.71 | 0.11 |
| Season (S) | 0.51 | 0.48 | 0.54 | 0.47 | 1.13 | 0.30 |
| D*R | 2.11 | 0.15 | 2.71 | 0.11 | 1.51 | 0.23 |
| D*S | 0.86 | 0.36 | 0.43 | 0.52 | 0.54 | 0.47 |
| R*S | 0.12 | 0.73 | 0.00 | 0.96 | 0.22 | 0.64 |
| D*R*S | 0.11 | 0.74 | 0.01 | 0.94 | 0.19 | 0.66 |

Notes: Three-way ANOVA was used to test the effects of duration (D), rate (R), season (S) of N addition and their interactions on soil fungal taxonomic and phylogenetic α-diversity. Significant effect (*p* < 0.05) is highlighted in bold.

**TABLE S2 |** Responses of soil physiochemical properties (average ± standard error, n = 12) to N addition at the short- and long-term sites

|  |  | SWC  (%) | pH | NH_4_^+^-N  (mg kg^-1^) | NO_3_^-^-N  (mg kg^-1^) | TOC  (%) | TN  (%) | C/N | TP  (%) | DOC  (mg kg^-1^) | DON  (mg kg^-1^) |
| --- | --- | --- | --- | --- | --- | --- | --- | --- | --- | --- | --- |
| Short-term | Control | 35.32±1.24 | 3.99±0.03 | 1.7±0.19 | 5.62±0.64 | 2.78±0.13 | 0.17±0.01 | 16.01±0.56 | 0.02 | 186±15.76 | 33.22±1.8 |
|  | N addition | 33.15±1.18 | 3.89±0.03 | 2.89±0.54 | 8.78±1.19 | 2.99±0.18 | 0.19±0.01 | 15.46±0.56 | 0.02 | 187.13±15.78 | 36.07±2.29 |
|  | *p* | 0.22 | **0.01** | 0.21 | **0.03** | 0.35 | 0.08 | 0.5 | 0.77 | 0.99 | 0.34 |
| Long-term | Control | 30.19±1.61 | 3.97±0.03 | 1.66±0.36 | 7.15±0.98 | 2.66±0.08 | 0.17±0.01 | 16.22±0.97 | 0.02 | 171.74±15.01 | 34.1±3.35 |
|  | N addition | 30.98±1.58 | 3.87±0.03 | 2.79±0.63 | 9.51±0.66 | 2.6±0.09 | 0.17±0.01 | 14.84±0.26 | 0.02 | 160.2±14.54 | 34.62±1.76 |
|  | *p* | 0.73 | **0.03** | 0.25 | 0.06 | 0.65 | 0.89 | 0.18 | 0.48 | 0.59 | 0.89 |

Notes: The *P* values detected from Student's test are listed. Numbers in bold indicate the significant differences (*p* < 0.05).

Abbreviation: SWC, soil water content; NH_4_^+^-N, ammonium N; NO_3_^-^-N, nitrate N; TOC, total organic carbon; TN, total N; C/N, total organic carbon / total N; TP, total phosphorus; DOC, dissolved organic carbon; DON, dissolved organic N.

**TABLE S3 |** ANOVA of environmental factors correlate with soil fungal taxonomic (based on Bray-curits distance) and phylogenetic (based on βMNTD) community composition at the short- and long-term sites

|  | *Factor* | *df* | *Variance* | *Pseudo-F* | *p-value* |
| --- | --- | --- | --- | --- | --- |
| Bray-curits | pH | 1 | 0.557 | 2.5326 | 0.001 |
|  | DON | 1 | 0.3993 | 1.8157 | 0.001 |
|  | DOC | 1 | 0.305 | 1.387 | 0.02 |
|  | NO_3_^-^-N | 1 | 0.2699 | 1.2272 | 0.048 |
|  | SWC | 1 | 0.232 | 1.0549 | 0.28 |
|  | TOC | 1 | 0.2381 | 1.0828 | 0.211 |
|  | C/N | 1 | 0.2308 | 1.0494 | 0.293 |
|  | TP | 1 | 0.2185 | 0.9934 | 0.442 |
|  | NH_4_^+^-N | 1 | 0.197 | 0.8957 | 0.803 |
|  | Residual | 38 | 8.3572 |  |  |
|  |  |  |  |  |  |
| βMNTD | pH | 1 | 0.13107 | 1.7925 | 0.001 |
|  | NO_3_^-^-N | 1 | 0.08566 | 1.1715 | 0.046 |
|  | DON | 1 | 0.08473 | 1.1588 | 0.058 |
|  | TOC | 1 | 0.08363 | 1.1438 | 0.083 |
|  | SWC | 1 | 0.0712 | 0.9737 | 0.585 |
|  | DOC | 1 | 0.07331 | 1.0026 | 0.421 |
|  | NH_4_^+^-N | 1 | 0.0719 | 0.9833 | 0.516 |
|  | C/N | 1 | 0.07706 | 1.0538 | 0.211 |
|  | TP | 1 | 0.07206 | 0.9855 | 0.542 |
|  | Residual | 38 | 2.7786 |  |  |

Abbreviation: SWC, soil water content (%); NH_4_^+^-N, ammonium N (mg kg^-1^); NO_3_^-^-N, nitrate N (mg kg^-1^); TOC, total organic carbon (%); TN, total N (%); C/N, total organic carbon / total N; TP, total phosphorus (%); DOC, dissolved organic carbon (mg kg^-1^); DON, dissolved organic N (mg kg^-1^).

**TABLE S4 |** Summary of the basic taxonomies and the network connectivity of the putative keystone species in control network at the short-term site

| Network category | ID | Classification | | | | | No. module | Connectivity |
| --- | --- | --- | --- | --- | --- | --- | --- | --- |
|  |  | Phylum | Class | Order | Family | Genus |  |  |
| Module hubs | OTU_967 | *Basidiomycota* | *Agaricomycetes* | *Sebacinales* | Unassigned | Unassigned | 0 | 6 |
|  | OTU_61 | *Basidiomycota* | *Agaricomycetes* | *Trechisporales* | Unassigned | Unassigned | 6 | 12 |
|  | OTU_477 | *Ascomycota* | Unassigned | Unassigned | Unassigned | Unassigned | 2 | 6 |
|  | OTU_344 | *Basidiomycota* | *Agaricomycetes* | *Thelephorales* | *Thelephoraceae* | *Tomentella* | 3 | 7 |
|  | OTU_66 | *Ascomycota* | *Eurotiomycetes* | *Chaetothyriales* | *Herpotrichiellaceae* | *Cladophialophora* | 5 | 7 |
|  | OTU_44 | *Ascomycota* | *Eurotiomycetes* | *Eurotiales* | *Trichocomaceae* | *Sagenomella* | 10 | 8 |
|  | OTU_11 | *Mortierellomycota* | *Mortierellomycetes* | *Mortierellales* | *Mortierellaceae* | *Mortierella* | 1 | 6 |
| Connectors | OTU_140 | *Ascomycota* | *Eurotiomycetes* | Unassigned | Unassigned | Unassigned | 4 | 5 |

**TABLE S5 |** Summary of the basic taxonomies and the network connectivity of the putative keystone species in N addition network at the short-term site

| Network category | ID | Classification | | | | | No. module | Connectivity |
| --- | --- | --- | --- | --- | --- | --- | --- | --- |
|  |  | Phylum | Class | Order | Family | Genus |  |  |
| Module hubs | OTU_3025 | *Ascomycota* | Unassigned | Unassigned | Unassigned | Unassigned | 10 | 8 |
|  | OTU_706 | *Ascomycota* | *Eurotiomycetes* | *Eurotiales* | Unassigned | Unassigned | 3 | 12 |
|  | OTU_439 | *Ascomycota* | *Dothideomycetes* | *Pleosporales* | *Didymosphaeriaceae* | Unassigned | 9 | 11 |
|  | OTU_27 | *Basidiomycota* | *Agaricomycetes* | *Boletales* | *Boletaceae* | Unassigned | 2 | 11 |
|  | OTU_63 | *Ascomycota* | *Sordariomycetes* | *Hypocreales* | *Hypocreaceae* | *Hypocrea* | 2 | 13 |
|  | OTU_1014 | *Ascomycota* | *Eurotiomycetes* | *Chaetothyriales* | *Herpotrichiellaceae* | *Cladophialophora* | 17 | 8 |
|  | OTU_135 | *Ascomycota* | *Sordariomycetes* | *Ophiostomatales* | *Ophiostomataceae* | *Ophiostoma* | 6 | 11 |
|  | OTU_87 | *Ascomycota* | *Sordariomycetes* | *Hypocreales* | *Hypocreaceae* | *Hypomyces* | 5 | 7 |
|  | OTU_73 | *Ascomycota* | *Dothideomycetes* | *Venturiales* | Unassigned | Unassigned | 4 | 13 |
| Connectors | OTU_470 | *Ascomycota* | *Sordariomycetes* | Unassigned | Unassigned | Unassigned | 1 | 4 |
|  | OTU_874 | *Ascomycota* | *Sordariomycetes* | *Chaetosphaeriales* | *Chaetosphaeriaceae* | Unassigned | 0 | 4 |
|  | OTU_140 | *Ascomycota* | *Eurotiomycetes* | Unassigned | Unassigned | Unassigned | 8 | 4 |
|  | OTU_240 | *Ascomycota* | *Leotiomycetes* | *Helotiales* | Unassigned | Unassigned | 11 | 4 |
|  | OTU_115 | Unassigned | Unassigned | Unassigned | Unassigned | Unassigned | 7 | 3 |
|  | OTU_7 | *Mortierellomycota* | *Mortierellomycetes* | *Mortierellales* | *Mortierellaceae* | *Mortierella* | 3 | 5 |

**TABLE S6 |** Summary of the basic taxonomies and the network connectivity of the putative keystone species in control network at the long-term site

| Network category | ID | Classification | | | | | No. module | Connectivity |
| --- | --- | --- | --- | --- | --- | --- | --- | --- |
|  |  | Phylum | Class | Order | Family | Genus |  |  |
| Module hubs | OTU_2359 | Unassigned | Unassigned | Unassigned | Unassigned | Unassigned | 9 | 6 |
|  | OTU_302 | *Ascomycota* | *Dothideomycetes* | *Venturiales* | Unassigned | Unassigned | 4 | 5 |
|  | OTU_295 | *Ascomycota* | *Sordariomycetes* | *Xylariales* | Unassigned | Unassigned | 1 | 14 |
|  | OTU_183 | *Ascomycota* | Unassigned | Unassigned | Unassigned | Unassigned | 11 | 11 |
|  | OTU_276 | *Basidiomycota* | *Agaricomycetes* | *Russulales* | *Russulaceae* | *Russula* | 4 | 6 |
|  | OTU_12 | *Basidiomycota* | *Agaricomycetes* | *Russulales* | *Russulaceae* | *Russula* | 3 | 10 |
| Connectors | OTU_737 | *Ascomycota* | *Eurotiomycetes* | Unassigned | Unassigned | Unassigned | 4 | 4 |
|  | OTU_359 | *Basidiomycota* | *Agaricomycetes* | *Sebacinales* | *Sebacinaceae* | *Sebacina* | 1 | 3 |
|  | OTU_3153 | *Ascomycota* | Unassigned | Unassigned | Unassigned | Unassigned | 11 | 5 |
|  | OTU_539 | *Rozellomycota* | *Rozellomycota_cls_Incertae_sedis* | *GS11* | Unassigned | Unassigned | 2 | 4 |
|  | OTU_223 | *Ascomycota* | *Leotiomycetes* | Unassigned | Unassigned | Unassigned | 11 | 6 |
|  | OTU_84 | *Ascomycota* | *Leotiomycetes* | *Helotiales* | *Myxotrichaceae* | *Oidiodendron* | 4 | 3 |
|  | OTU_66 | *Ascomycota* | *Eurotiomycetes* | *Chaetothyriales* | *Herpotrichiellaceae* | *Cladophialophora* | 1 | 3 |
|  | OTU_85 | *Ascomycota* | *Sordariomycetes* | Unassigned | Unassigned | Unassigned | 4 | 4 |
|  | OTU_4 | *Basidiomycota* | *Tremellomycetes* | *Tremellales* | *Trimorphomycetaceae* | *Saitozyma* | 0 | 13 |
| Network hub | OTU_73 | *Ascomycota* | *Dothideomycetes* | *Venturiales* | Unassigned | Unassigned | 2 | 18 |

**TABLE S7 |** Summary of the basic taxonomies and the network connectivity of the putative keystone species in N addition network at the long-term site

| Network category | ID | Classification | | | | | No. module | Connectivity |
| --- | --- | --- | --- | --- | --- | --- | --- | --- |
|  |  | Phylum | Class | Order | Family | Genus |  |  |
| Module hubs | OTU_1359 | *Ascomycota* | *Sordariomycetes* | *Hypocreales* | *Cordycipitaceae* | *Lecanicillium* | 1 | 12 |
|  | OTU_703 | Unassigned | Unassigned | Unassigned | Unassigned | Unassigned | 1 | 15 |
|  | OTU_2555 | *Ascomycota* | *Sordariomycetes* | Unassigned | Unassigned | Unassigned | 2 | 7 |
|  | OTU_353 | *Ascomycota* | *Eurotiomycetes* | *Chaetothyriales* | Unassigned | Unassigned | 1 | 13 |
|  | OTU_147 | *Ascomycota* | *Leotiomycetes* | *Helotiales* | *Hyaloscyphaceae* | Unassigned | 0 | 38 |
|  | OTU_308 | *Rozellomycota* | Unassigned | Unassigned | Unassigned | Unassigned | 9 | 6 |
|  | OTU_47 | Unassigned | Unassigned | Unassigned | Unassigned | Unassigned | 6 | 7 |
|  | OTU_39 | *Basidiomycota* | *Agaricomycetes* | *Agaricales* | *Hydnangiaceae* | *Laccaria* | 0 | 38 |
|  | OTU_158 | *Ascomycota* | *Eurotiomycetes* | *Chaetothyriales* | *Herpotrichiellaceae* | *Cladophialophora* | 8 | 15 |
|  | OTU_86 | *Ascomycota* | Unassigned | Unassigned | Unassigned | Unassigned | 5 | 6 |
|  | OTU_385 | *Ascomycota* | *Eurotiomycetes* | Unassigned | Unassigned | Unassigned | 2 | 7 |
|  | OTU_28 | *Basidiomycota* | *Agaricomycetes* | *Russulales* | *Russulaceae* | Unassigned | 0 | 43 |
|  | OTU_3 | *Basidiomycota* | *Agaricomycetes* | *Russulales* | *Russulaceae* | *Lactarius* | 7 | 11 |
| Connectors | OTU_1020 | *Ascomycota* | *Eurotiomycetes* | *Eurotiales* | *Aspergillaceae* | *Penicillium* | 2 | 3 |
|  | OTU_315 | *Basidiomycota* | *Agaricomycetes* | *Agaricales* | *Tricholomataceae* | Unassigned | 2 | 5 |
|  | OTU_670 | Unassigned | Unassigned | Unassigned | Unassigned | Unassigned | 2 | 3 |
|  | OTU_175 | *Ascomycota* | Unassigned | Unassigned | Unassigned | Unassigned | 2 | 3 |
|  | OTU_464 | *Ascomycota* | Unassigned | Unassigned | Unassigned | Unassigned | 8 | 4 |
|  | OTU_5094 | *Ascomycota* | *Leotiomycetes* | Unassigned | Unassigned | Unassigned | 3 | 8 |

**SUPPLEMENTARY FIGURES**


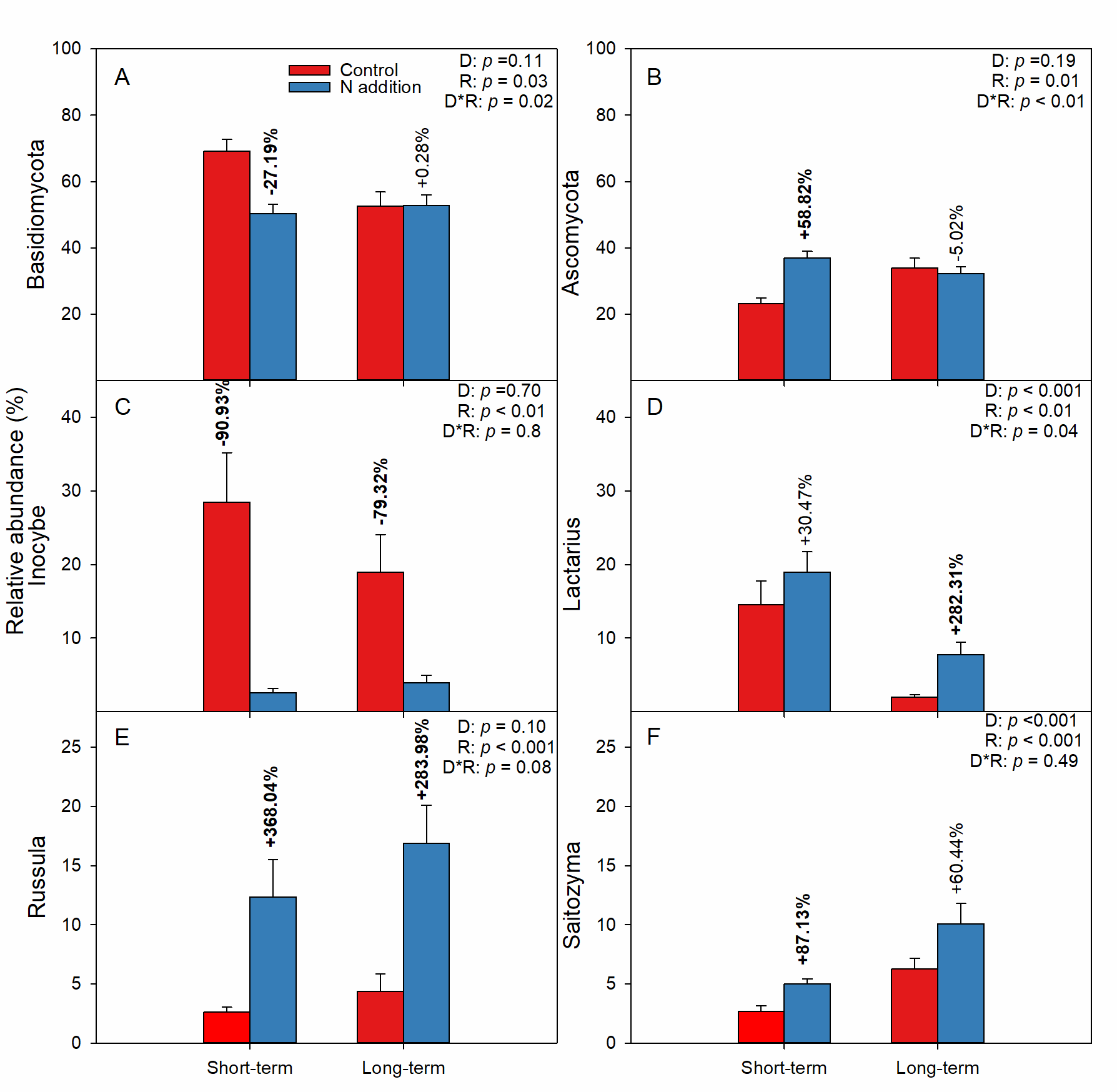


**FIGURE S1 |** The varaitions of relative abundance of major phyla **(A, B)**, genera **(C-F)** under N addition are indicated by student's t-test at the short- and long-term sites, respectively. The insert texts indicate the effects of duration (D), rate (R) of N addition and their interactions on the relative abundance of fungal major phyla and genera detected by two-way ANOVA. The bold numbers denote the significantly differences (*p* < 0.05) of aforementioned parameters between control and N addition at each site.


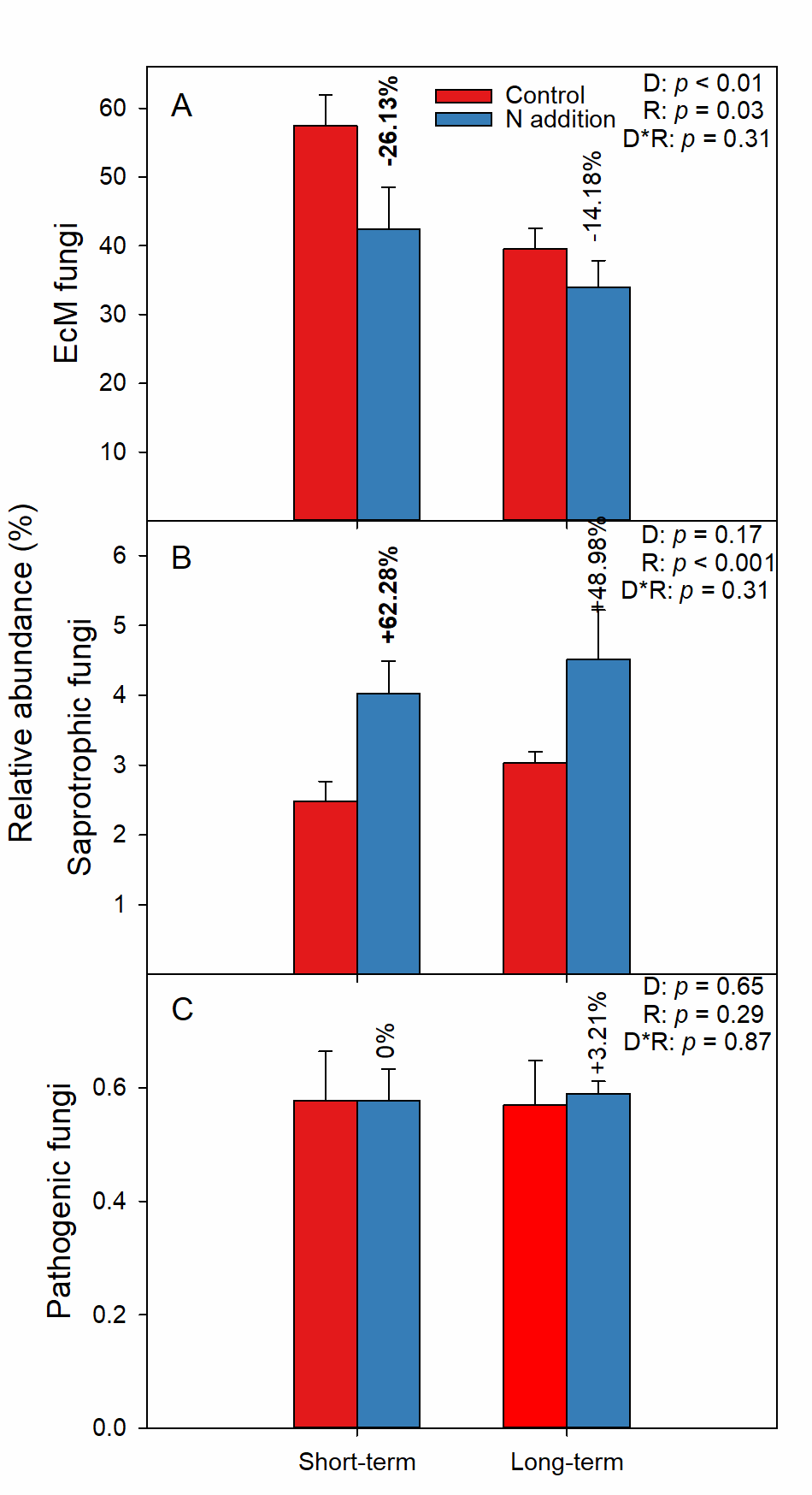


**FIGURE S2 |** The varaitions of relative abundance of major funtional guilds under N addition are indicate by student's t-test at the short- and long-term sites, respectively. The insert texts indicate the effects of duration (D), rate (R) of N addition and their interactions on the relative abundance of fungal major funtional guilds detected by two-way ANOVA. The bold numbers denote the significantly differences (*p* < 0.05) of major funtional guilds between control and N addition at each site.
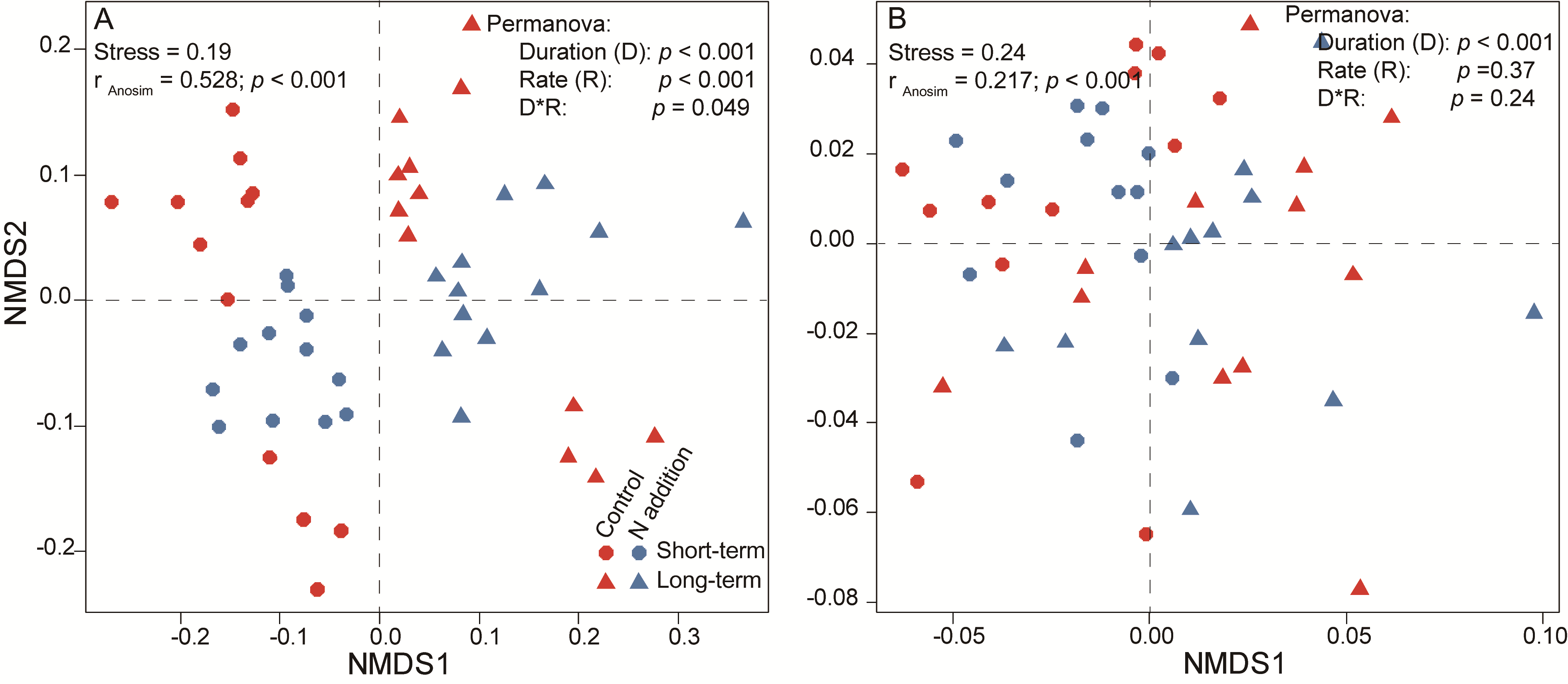
**FIGURE S3 |** Non-metric multidimensional scaling (NMDS) plots based on Bray-Curtis distance **(A)** and βMNTD **(B)** of fungal community. The results of Permanova show in the graph to reveal the effects of duration (D), rate (R) of N addition and their interactions on taxonomic and phylogenetic community dissimilarity.


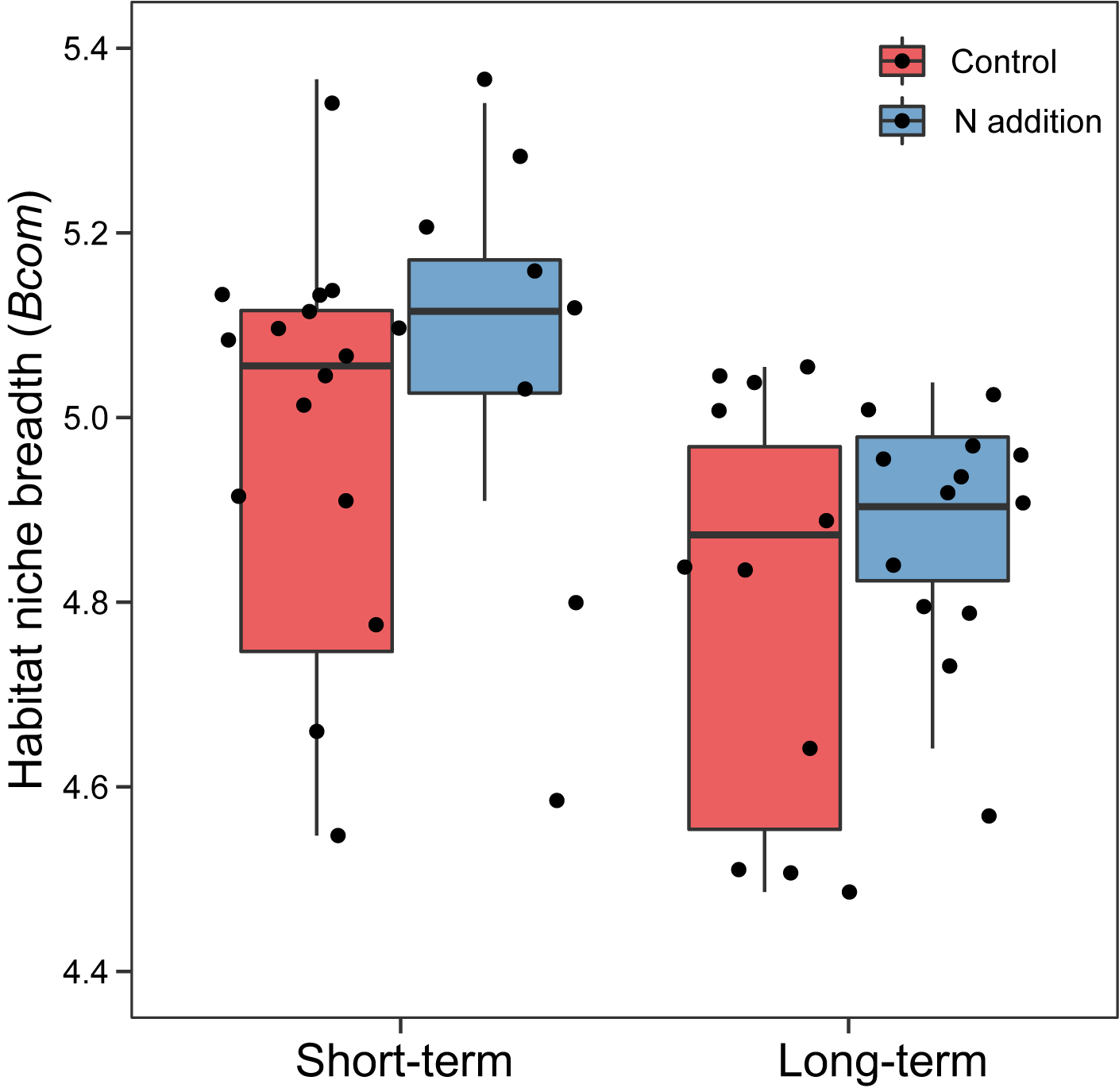


**FIGURE S4 |** Comparison of mean habitat niche breadth (*Bcom*) in all taxa of fungal community between control and N addition at the short- and long-term sites, respectively.


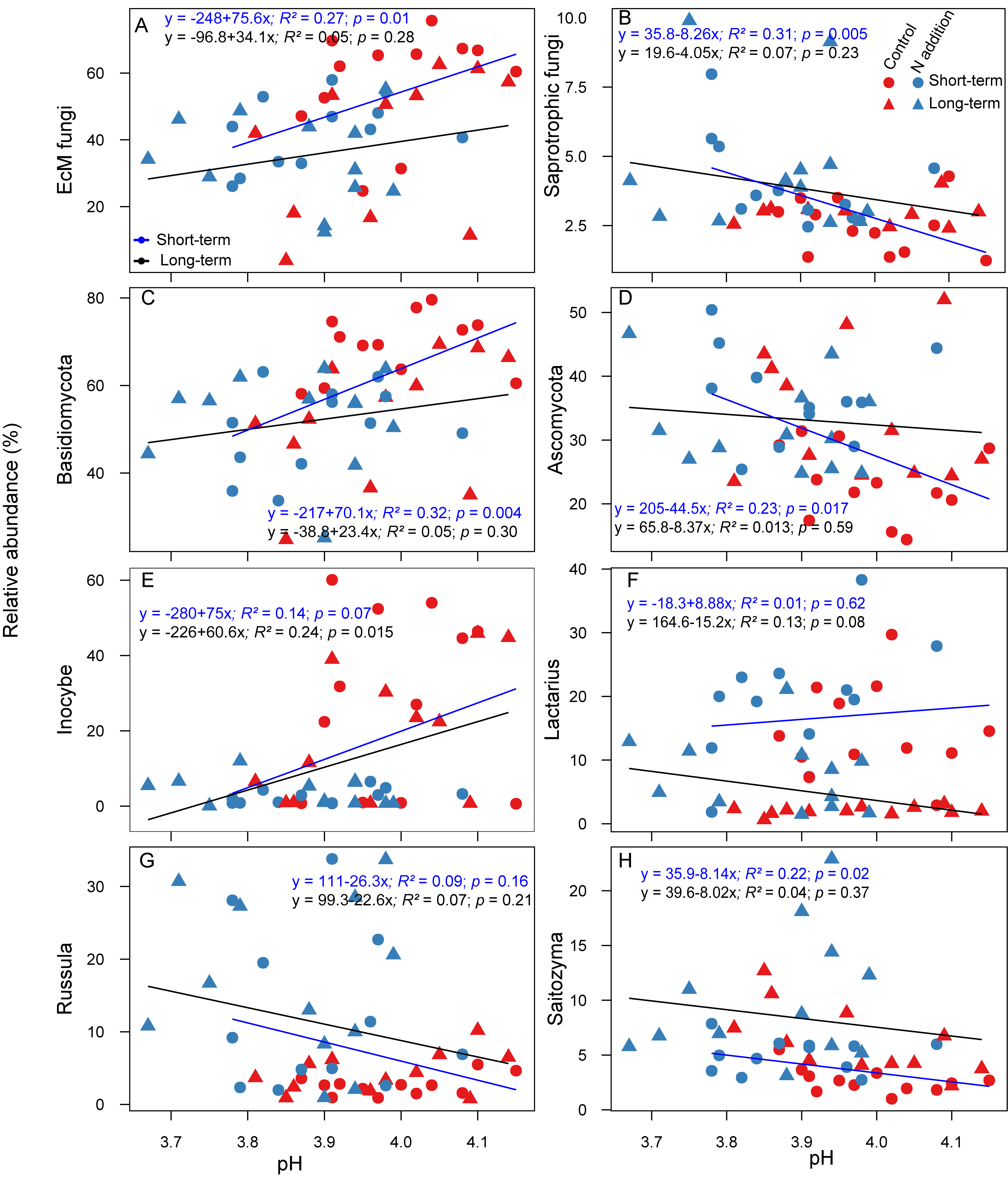


**FIGURE S5 |** Relationships between soil pH and the relative abundance of major functional guilds **(A, B)**, phyla **(C, D)** and genera **(E-H)** are indicated by linear regressions model.


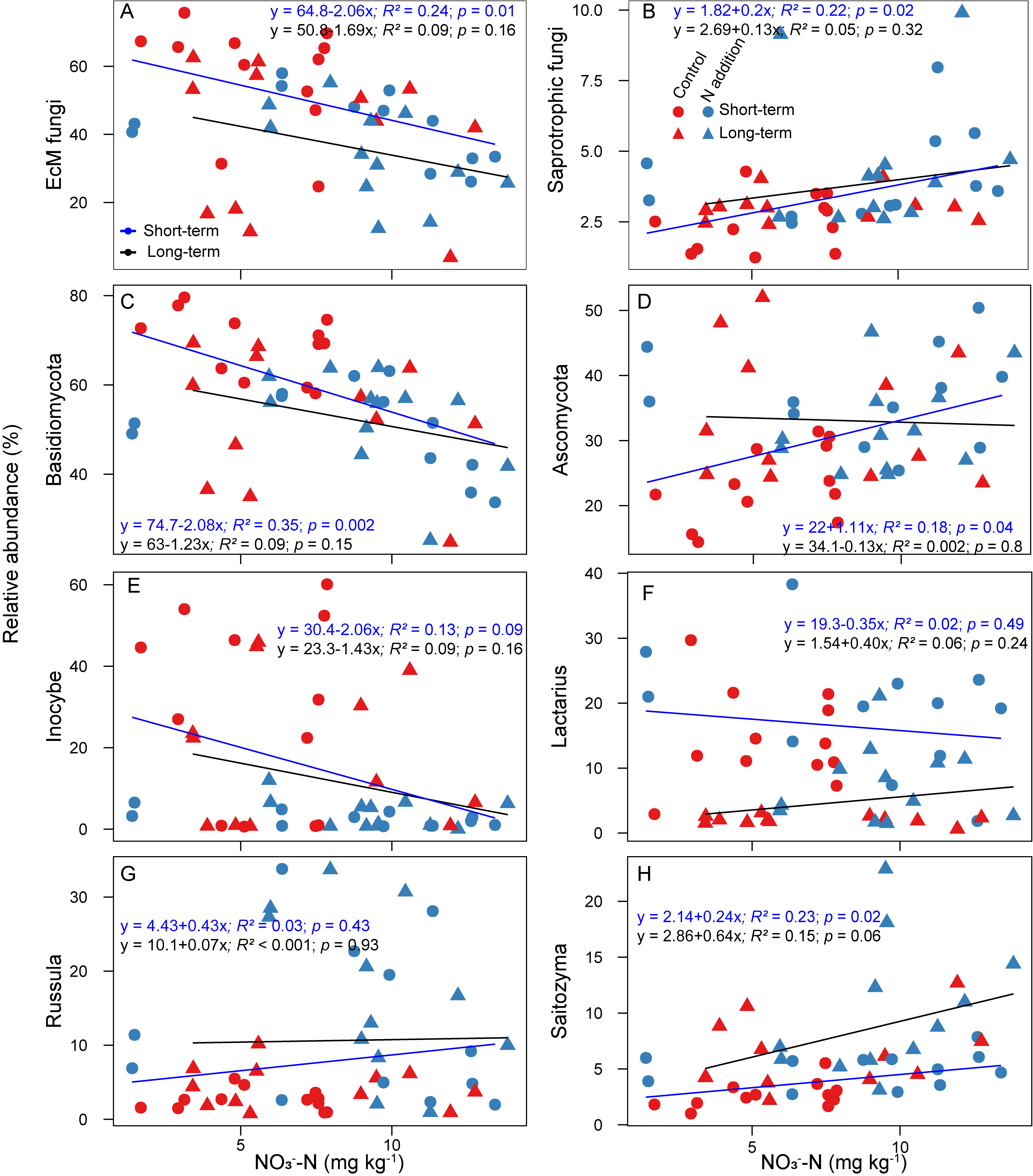


**FIGURE S6 |** Relationships between the concentration of NO_3_^-^-N and the relative abundance of major functional guilds **(A, B)**, phyla **(C, D)** and genera **(E-H)** are indicated by linear regressions model.


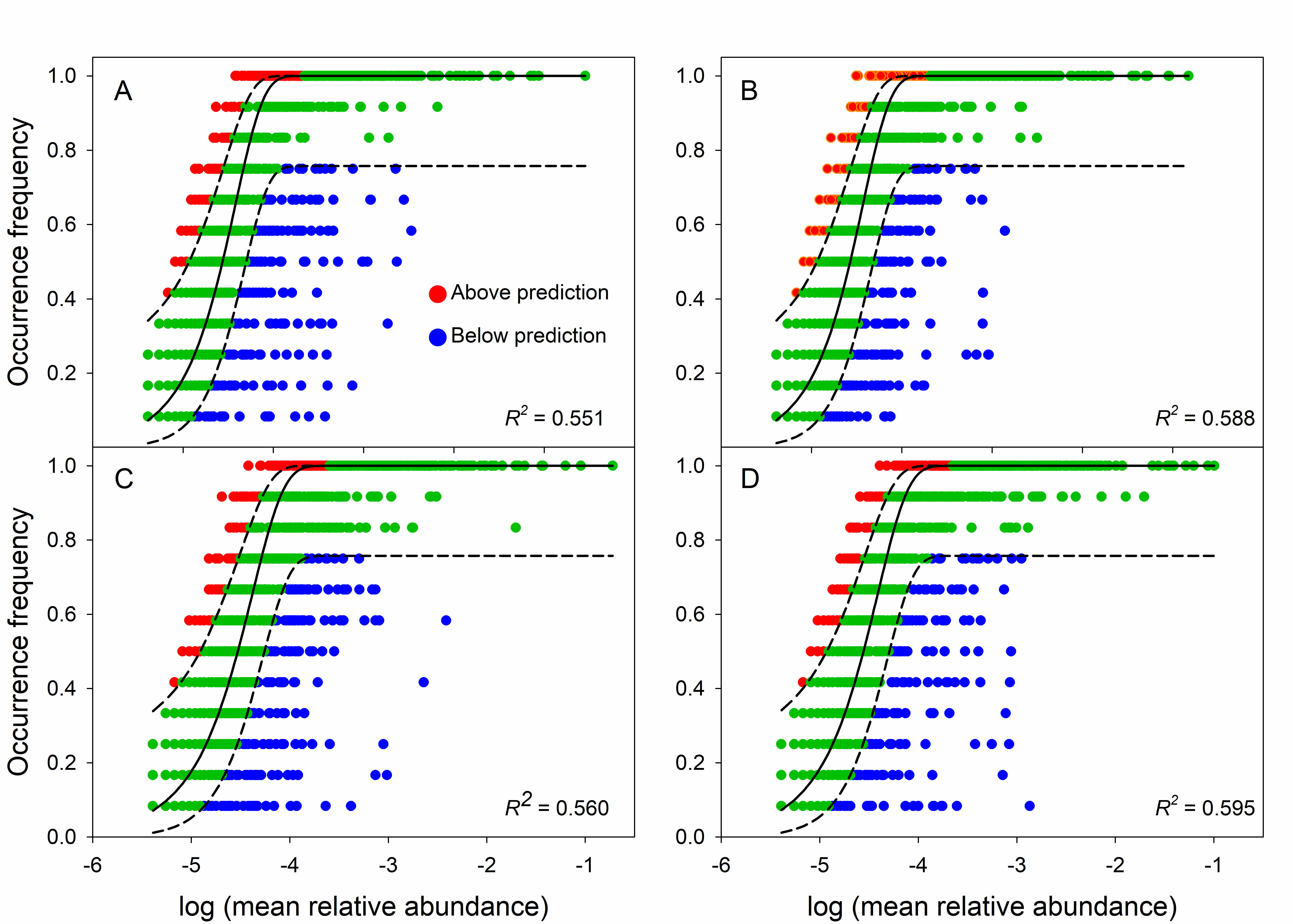
**FIGURE S7 |** Fit of the neutral community model of community assembly for control **(A, C)** and N addition **(B, D)** at the short- and long-term sites, respectively. OTUs that occurred more frequently than predicted by the model are shown in red, while those that occurred less frequently than predicted are shown in blue. OTUs that occurred within prediction are shown in green. Dashed lines represent 95% confidence intervals around the model prediction (black line). *R^2^* indicate the fit to this model.
